# Application of failure mode and effects analysis (FMEA) to improve medication safety in the dispensing process – a study at a teaching hospital, Sri Lanka

**DOI:** 10.1186/s12889-021-11369-5

**Published:** 2021-07-20

**Authors:** J. A. L. Anjalee, V. Rutter, N. R. Samaranayake

**Affiliations:** 1 Colombo South Teaching Hospital, Kalubowila, Dehiwala, Sri Lanka; 2 Faculty of Graduate Studies, University of Sri Jayewardenepura, Gangodawila, Nugegoda, Sri Lanka; 3 Commonwealth Pharmacists Association, London, UK; 4 Department of Pharmacy and Pharmaceutical Sciences, Faculty of Allied Health Sciences, University of Sri Jayewardenepura, Gangodawila, Nugegoda, Sri Lanka

**Keywords:** Failure mode and effects analysis, FMEA, Pharmacists, Dispensing process, Sri Lanka

## Abstract

**Background:**

Failure mode and effects analysis (FMEA) is a prospective, team based, structured process used to identify system failures of high risk processes before they occur. Medication dispensing is a risky process that should be analysed for its inherent risks using FMEA. The objective of this study was to identify possible failure modes, their effects, and causes in the dispensing process of a selected tertiary care hospital using FMEA.

**Methods:**

Two independent teams (Team A and Team B) of pharmacists conducted the FMEA for two months in the Department of Pharmacy of a selected teaching hospital, Colombo, Sri Lanka. Each team had five meetings of two hours each, where the dispensing process and sub processes were mapped, and possible failure modes, their effects, and causes, were identified. A score for potential severity (S), frequency (F) and detectability (D) was assigned for each failure mode. Risk Priority Numbers (RPNs) were calculated (RPN=SxFxD), and identified failure modes were prioritised.

**Results:**

Team A identified 48 failure modes while Team B identified 42. Among all 90 failure modes, 69 were common to both teams. Team A prioritised 36 failure modes, while Team B prioritised 30 failure modes for corrective action using the scores. Both teams identified overcrowded dispensing counters as a cause for 57 failure modes. Redesigning of dispensing tables, dispensing labels, the dispensing and medication re-packing processes, and establishing a patient counseling unit, were the major suggestions for correction.

**Conclusion:**

FMEA was successfully used to identify and prioritise possible failure modes of the dispensing process through the active involvement of pharmacists.

## Background

Medication safety is a global concern and a matter of interest for healthcare professionals and researchers worldwide. As a result, in 2017, the World Health Organization (WHO) initiated the “Third Global Patient Safety Challenge with a theme on medication safety” along with the challenge to “reduce the frequency and impact of medication errors” [1]. The National Coordinating Council for medication error reporting and prevention defines medication errors as “any preventable event that may cause or lead to inappropriate medication use or patient harm while the medication is in the control of the healthcare professional, patient, or consumer” [2]. Medication errors are further classified as prescribing, dispensing, and medication administration errors. If any failure in communication occurs at prescribing or dispensing in ambulatory care it will further result in patient compliance errors [2]. It is reported that these errors are caused mainly due to faulty systems and rarely due to human neglect [1].

WHO estimates that the annual cost of medication errors is around US$42 billion [1]. It is estimated that National Health Service (NHS) of the United Kingdom spends £1 billion per annum as extra hospitalisation costs due to preventable adverse effects [3]. Gathering the knowledge from various regions of the world, WHO demonstrates that older patients (> 75 years), patients with poly-pharmacy, and patients at transition of care (either discharge after hospitalisation or transfer from primary care to secondary care) are most vulnerable for medication errors [4]. Studies on medication errors have been reported from countries of different regions of the world such as the United Kingdom, Saudi Arabia, Sweden, and Mexico [4]. These findings demonstrate that medication errors are a global issue and highlight the importance of addressing the issue through research and other scientific moves.

The focus of the present study was on dispensing errors. Dispensing is an important element of pharmaceutical care, which in turn is an indispensable aspect of total patient care. As the American Pharmaceutical Association describes, the pharmacist must be “responsible for the appropriate use of medications, devices, and services to achieve optimal therapeutic outcomes” [5] and must be responsible for ensuring patient safety. Especially in ambulatory care, a pharmacist dispensing medication is the last healthcare professional and any error that takes place in this step directly reaches to the patient [6]. A systematic review on dispensing errors reports that dispensing error rate varies from 0.015 to 33.5% [7]. Concerning the error type, dispensing the wrong medication was the most common one. Other identified frequent error types were dispensing wrong medication strength, and wrong dosage form. “High workload, low staffing, mixing of Look Alike Sound Alike (LASA) medications, issues in knowledge/experience, distractions, and communication issues” were identified as common reasons of dispensing errors [7]. In United Kingdom, ~ 17% of reported medication errors were related to dispensing [7]. It is reported that 37% of dispensing errors are organisational or system problems while 30% are related to the individual professional, 17% to prescription, 10% to medication, and 4% to the patient [8]. Thus, proactive efforts taken to prior identification of possible failures of the dispensing process would clearly benefit in improving patient safety.

Various Human Reliability Analysis (HRA) methods are available to identify errors and weaknesses in systems. HRA techniques aim to identify failures of systems and people involved but without blaming or shaming [3]. The three approaches of HRA methods include retrospective, prospective and on-line analysis methods [9]. Most HRA methods are of commercial origin and are not found in scientific literature. A review of HRA techniques states that methods like CREAM (Cognitive Reliability and Error Analysis), MORT (Management Oversight Risk Trees Method) and THERP (Human Error Rate Prediction) methods are still not used in healthcare [10]. Historically, Root Cause Analysis (RCA) was the commonly used method to find out root causes of errors that occurred in the healthcare sector. RCA is a means of identifying WHAT, HOW and WHY an event occurred [11].

The National Academy of Medicine recommends conducting prospective risk analysis studies on medication safety in pharmacy rather than basic epidemiological studies [12]. The Failure Mode and Effects Analysis (FMEA) is an ideal tool for this purpose as it is able to identify potential failures before harmful events occur [13]. FMEA offers a proactive approach to detecting failures in contrast to incident analysis and Root Cause Analysis which are performed retrospectively. As FMEA is able to identify errors before it happens, industries such as aviation, aerospace, nuclear power and automobiles [14] use it widely. Lately, FMEA has been adopted to assess risks in healthcare and to identify areas that need improvement in the healthcare system. The United Kingdom National Patient Safety Agency recommends to apply FMEA to assess new policies and procedures before implementing them [15] and the Joint Commission, USA has asked its accredited institutes to carry out an annual proactive risk assessment study such as FMEA [15, 16].

FMEA is used in many healthcare specialties including chemotherapy [17–20], paediatrics [18, 21–23], and pharmacy, and in different settings such as in-patient settings [16–18, 24], intensive care units [23, 25], community clinics [26], and community pharmacies [12, 27]. FMEA has even been successfully used to analyse new policies before implementing them [28]. However, there were no reports on using FMEA to assess the safety of the dispensing process of out-patients in hospitals.

FMEA is a systematic and step-wise procedure starting with selecting a clearly defined process to assess and assemble a multidisciplinary team. Afterwards processes and sub processes of the selected process are mapped using the team’s collective knowledge and by focusing on key components of the process. After mapping the process, the team does a brainstorming to identify potential failure modes for each sub process. Then team identifies the effects and causes of potential failure modes and enters the results into the spreadsheet. Professional knowledge and personal experience of team members and information from literature is useful in this step. The team then prioritises the potential failure modes, considering the severity, frequency and detectability of failure modes. Finally the team redesigns or modifies the process to avoid or minimise the failures [13, 29].

With the intention of having a comprehensive understanding on FMEA prior to starting the present study, authors conducted a systematic review on application of FMEA on different medication use processes. PubMed, JSTOR, Emerald, SAGE, Wiley online, Oxford journals, Web of science, Scopus and Cochrane library databases were searched for relevant studies from January 2006 to December 2017 [30]. During this review, we found a number of studies using FMEA in areas such as chemotherapy [17–20], parenteral nutrition [31], medication management [2, 32, 33], medication administration [34, 35], medication use process (one or more steps from prescribing to dispensing) among in-patients [24, 36, 37] and paediatrics [18, 21–23, 38]. We found only two studies [12, 27] using FMEA to analyse the dispensing process of out-patients where both studies were carried out in the community. There were no reports on FMEA carried out on the dispensing process for ambulatory patients in hospitals. So, to the best of our knowledge, this study is the first model for using FMEA in an out-patient hospital pharmacy to analyse the safety of the dispensing process.

Therefore, the objective of this study was to identify possible failures in the dispensing processes serving out-patients of a tertiary care hospital, their effects, and causes, using failure mode and effects analysis and to recommend corrective actions for selected failure modes.

## Methodology

The present cross sectional descriptive study was conducted from August 2018 to October 2018 in the Pharmacy Department of a selected teaching hospital, Colombo, Sri Lanka. Selection of the hospital was based on convenience. The Pharmacy Department consists of four dispensing units for out-patients, one in-patient pharmacy, one surgical store and a main medication store. The FMEA process was carried out to assess the safety of the medication dispensing process for out-patients.

Approximately 2000 OPD (urgent care) patients and 2500 clinic patients per day utilise the Pharmacy Department, which is staffed by 15 pharmacists at the time of the study. The hospital uses hand-written prescriptions while medication stock management, dispensing and documentation are also manual. During the dispensing process, each prescription is handled by one pharmacist only. Selected high volume medications are pre-packed as monthly supplies for the ease of dispensing, labeled with the name and strength of the medication and are stored in separate drawers. One medication is packed into packets of different quantities according to the requirement (e.g. Metformin tablet packets with 56 tablets and 84 tablets). Some selected medications are considered as “Accountable medications” which require strict documentation and are determined according to national and institutional guidelines. Criteria for determination of accountable medications are given in “Manual on management of drugs” published by Ministry of Healthcare and Nutrition [39].

**Fig. 1:**
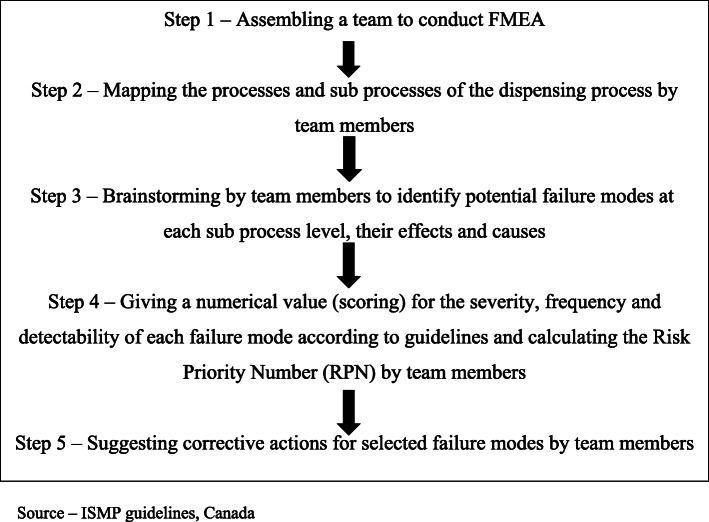
Steps of the Failure Mode and Effects Analysis (FMEA) according to ISMP, Canada guidelines [13]

FMEA was conducted according to guidelines specified in the FMEA framework of the Institute of Safe Medication Practices (ISMP), Canada [13] as illustrated in Fig. 1.

### Step 1 – assembling a team to conduct FMEA

Thirteen pharmacists participated in two teams for FMEA discussions. Pharmacists involved in dispensing medications as their daily routine of work and pharmacist in-charge of dispensing units were included in the study. Participants, after consenting to participate in the study, were divided in to two teams (Team A and Team B) to avoid any disruption to the daily dispensing process of the study hospital. This process is consistent with past studies where FMEA discussions were conducted in two or more teams to avoid practical issues such as better representation of participants and avoid disruption to patient services [15, 16, 38, 40]. Both teams followed the same set of steps and had a similar composition of team members. We ensured that each team had one pharmacist in-charge to represent the managerial level, at least one senior pharmacist with more than ten years of working experience, and at least one graduate pharmacist. Each team had five meetings of two hours each. The researcher participated as the facilitator for all the FMEA discussions conducted by both teams and all discussions were audio recorded.

At the first meeting the researcher introduced the FMEA process to team members with illustrations [13, 41] and re-emphasised on the importance of a safety culture. To ensure that all were knowledgeable about the concept of a safety culture in the hospital, all the team members previously (Five months prior to this FMEA) attended a workshop on medication safety organised by the research team where various aspects of medication safety were emphasised [42]. This effort indicated that members of both teams were knowledgeable on medication safety and safety culture before engaging in FMEA.

At the first meeting, all team members agreed that dispensing medications is a high-risk process highly likely to cause patient harm if any error occurred.

### Step 2 – mapping the process and sub processes of dispensing

Initially, each team member individually and independently sketched the main steps of the dispensing process as they perceived the workflow. Then team members collated individual inputs to map one final dispensing process, agreed by all team members, to be used in subsequent steps of the FMEA process. The team then identified and mapped sub processes for each dispensing step they had identified.

### Step 3 – brainstorming to identify potential failure modes in each sub process of dispensing, their effects and causes

In the next step, team members brainstormed and identified possible failure modes in each sub process of dispensing, and documented them as recommended by ISMP, Canada [13]. Each failure mode was given an identification number. Next, team members brainstormed to identify possible effects and causes of each failure mode. Disagreements were discussed until a final consensus was reached by team members.

### Step 4 – giving a numerical value (scoring) for the severity, frequency and detectability of each failure mode and calculating the risk priority number (RPN)

Each failure mode was scored separately for severity, frequency and detectability. Numerical scores were assigned by team members based on their perception using guidelines specified by ISMP, Canada FMEA framework [13] (Table 1).

**Table 1:** Scoring scale given by ISMP, Canada for severity, frequency and detectability of failure modes (Source – FMEA framework, ISMP, Canada [13])

	Definition	Score
Severity (S)	No effect *(Failure is not noticeable and does not affect the patient or process)*	1
	Slight effect *(Failure causes minor effects or is a trouble to the patient or process, without injury or increase in level of care required)*	2
	Moderate effect *(Failure causes some performance loss and may increase the level of care (*e.g.*, requiring hospitalisation or increasing the length of hospital stay)*	3
	Major effect *(Failure causes a high degree of performance loss, with permanent impact on the patient)*	4
	Severe or catastrophic effect *(Failure causes death or major, permanent loss of function)*	5
Frequency (F)	Yearly	1
	Monthly	2
	Weekly	3
	Daily	4
	Hourly	5
Detectability* (D)	Always	1
	Likely	2
	Unlikely	3
	Never	4

*Detectability was defined as ‘Detectability of the error before it reaches to the patient’

Team members came to a final consensus on scores given for each failure mode. Disagreements were resolved through discussion until 100% agreement was reached within each team. The three individual scores (score for severity, score for frequency, score for detectability for each failure mode) were multiplied to calculate the risk priority number (RPN = S x F x D) for each failure mode. According to the scale, the RPN ranged from 1 to 100. Failure modes that were not common to the two teams were exchanged for assigning scores.

### Step 5 – suggesting corrective actions for selected failure modes

Team members then recommended possible corrective actions for prioritised failure modes. Failure modes with high scores for severity or frequency or detectability (i.e. difficult to detect), having scoring values of three or higher (scoring guidelines – Table 1) were discussed for corrective measures. Failure modes with low RPN values and low severity scores were not discussed further. Team members then discussed on feasibility of suggested corrective actions and highlighted the most important and feasible ones.

A feedback was obtained from the participants at the end of the discussions using a questionnaire developed in-house. The components of the questionnaire were based on the previously published studies on FMEA.

## Results

### Step 1 – assembling a team to conduct FMEA

Team A had six female pharmacists including one in-charge pharmacist, one graduate pharmacist, two senior pharmacists with more than ten years of experience, and two junior pharmacists. Team B had four male and three female pharmacists including one in-charge pharmacist, two graduate pharmacists, two senior pharmacists with more than ten years of experience, and one junior pharmacist.

### Step 2 – mapping the process and sub processes of dispensing

**Fig. 2:**
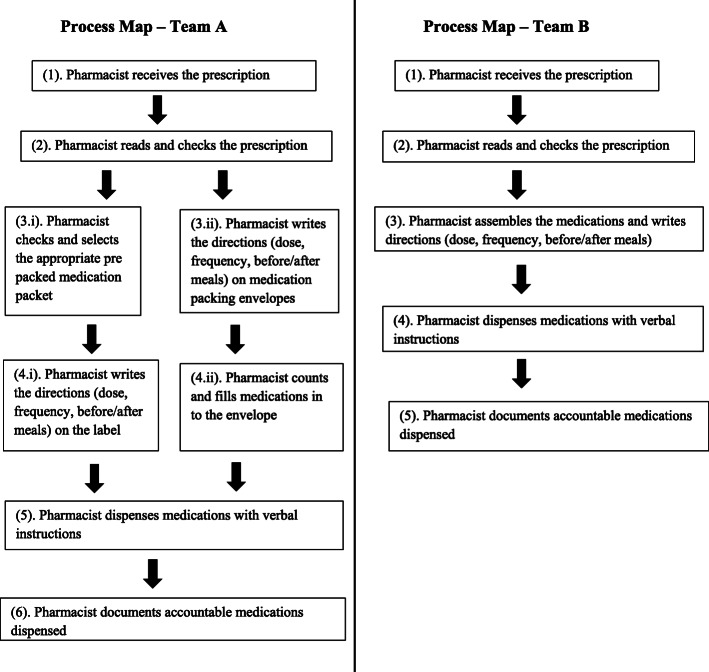
Dispensing process maps of Team A and Team B

**Fig. 3:**
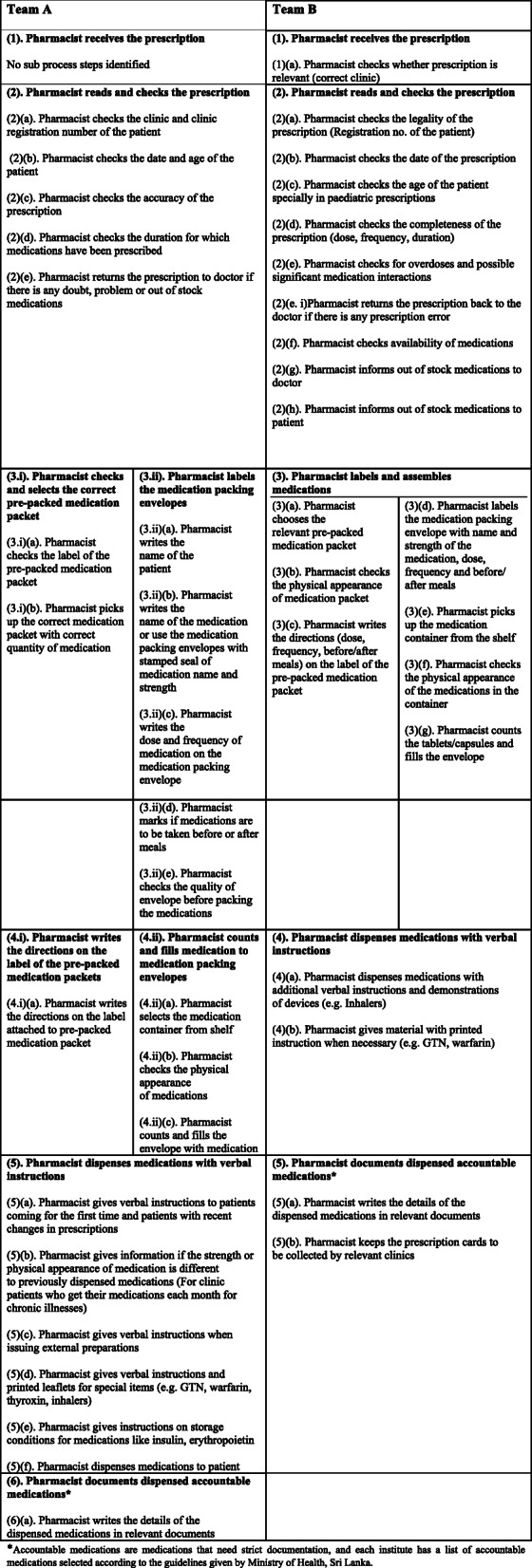
Sub processes of dispensing identified by Team A and Team B. *Accountable medications are medications that need strict documentation, and each institute has a list of accountable medications selected according to the guidelines given by Ministry of Health, Sri Lanka

Team A identified eight main process steps and 24 sub processes while Team B identified five main process steps and 21 sub processes. Process and sub process maps of both teams are shown in Figs. 2 and 3 respectively.

### Step 3 – brainstorming to identify potential failures of each sub process, their effects and causes

During the brain storming process, Team A identified 48 failure modes and Team B identified 42 failure modes. Among all 90 failure modes, 69 were common to both teams. Failure modes identified by both teams, and failure modes identified only by one specific team are shown in Table 2.

**Table 2:** Failure modes identified by Failure Mode and Effects Analysis and their Risk Priority Numbers

Common failure modes identified by both Team A and Team B		
Failure mode	RPN A	RPN B
1 Patient is issued a clinic prescription card belonging to another patient by mistake	20	6
2 Pharmacist dispenses medications to a clinic prescription that should have been dispensed at another clinic dispensing counter	8	8
3 Pharmacist does not check the clinic registration number of the patient	20	6
4 Pharmacist does not check the date of the prescription and age of the patient	16	24
5 Pharmacist misreads the medication name, dose or strength leading to wrong drug error when dispensing	15	16
6 Pharmacist unintentionally misses dispensation of some medications in long prescriptions	24	4
7 Pharmacist fails to identify prescribing errors on prescriptions	12	24
8 Pharmacist misreads the duration of the prescription leading to dispensation of the wrong quantity of medications	8	12
9 Pharmacist does not notify patient on out of stock medications	12	16
10 Pharmacist picks up the wrong medication packet (pre-packed) without checking the label	30	12
11 Pharmacist picks up the medication packet (pre-packed) with the wrong quantity	20	12
12 Pharmacist incompletely labels the medication packet having hand-written or partially hand-written labels	27	24
13 Pharmacist accidentally transcribes an incorrect dose or frequency to the medication label	36	6
14 Pharmacist writes directions (dose, frequency, before/after meals) in unclear handwriting	12	18
15 Pharmacist picks the wrong medication container from the dispensing shelf	8	8
16 Pharmacist does not check the physical appearance of medications in the container before preparation to assess colour and shape of medications for any decompositions	18	8
17 Pharmacist counts the wrong quantity of medications	40	16
18 Pharmacist fills the medications to a wrong envelope which was labelled for another medication	6	12
19 Patient does not understand the language of written instructions and/or verbal instructions given by the pharmacist	4	4
20 Pharmacist fails to tell some important information when giving verbal instructions briefly	12	12
21 Pharmacist gives incomplete instructions for external preparations and/or only give verbal instructions without written instructions (e.g. dermatological preparations)	18	12
22 Pharmacist fails to give verbal instructions	18	8
23 Leaflets may be unavailable and/or pharmacist may forget to give it to the patient	4	3
24 Pharmacist fails to document accountable medications	24	4
**Failure modes identified by Team A only (but scored by both teams)**		
25 Pharmacist incorrectly guesses information on unclear prescriptions	8	18
26 Pharmacist uses an envelope with an incomplete or unclear label stamp to pack medications	12	8
27 Pharmacist fails to check the quality of the medication packing envelope	15	2
28 Pharmacist fills the medications into an unlabeled medication packing envelope	12	8
29 Pharmacist fails to fill a labeled medication packing envelope	18	4
30 Leaflets may be unavailable in different languages (e.g. Tamil)	4	2
31 Pharmacist fails to dispense some filled medication packets to the patient	12	18
32 Pharmacist dispenses unfilled medication packets to the patient	12	4
33 Pharmacist dispenses or patient takes wrong medication packets which are left on the dispensing table	18	8
34 Pharmacist fails to update the accountable medication in manual log books daily	5	2
**Failure modes identified by Team B only (but scored by both teams)**		
35 Pharmacist accidentally mixes-up prescriptions of two paediatric patients from the same family	6	12
36 Pharmacist marks available medications as out of stock medications	1	12
37 Support staff (non-pharmacist) accidentally packs a wrong medication into pre-packed and sealed medication packets	12	12
38 Pre-packed medication packs may contain expired medications	9	9
39 Pre-packed medication packets may be left for longer duration after packing	6	8
40 Pharmacist gives only written medication directions to illiterate patients without verbal/pictorial communication	3	12
41 Pharmacist fails to check the expiry date of the medication	9	6
42 Pharmacist accidentally fills a wrong prescription given by another patient	3	6

*RPN A* Risk Priority Numbers assigned by Team A; *RPN B* Risk Priority Numbers assigned by Team B

Numbers of failure modes identified by each team at each process step are shown in Table 3.

**Table 3:** Number of failure modes identified for each step of the main process

Main process of dispensing	Number of identified failure modes	
	Team A (***N*** = 48)	Team B (***N*** = 42)
Pharmacist receives the prescription	1	2
Pharmacist checks the prescription	9	10
Pharmacist selects pre-packed medication packets with attached labels and writes instructions on them	5	12
Pharmacist labels medication packing envelopes and fills medications to them	13	9
Pharmacist dispenses medications with verbal instructions	14	6
Pharmacist documents details of accountable medications dispensed	6	3

Among sub processes, Team A identified the highest number of failure modes in checking the accuracy of prescriptions (*N* = 4), and in counting and filling medications into envelopes by pharmacists (*N* = 4). Team B identified the highest number of failure modes in sub processes, selecting the medication pack to be dispensed from the pre-packed medication tray (*N* = 5), and writing directions on label (*N* = 5).

Among the effects and causes of identified failure modes, the ones common to both teams are indicated in Table 4.

**Table 4:** Effects and causes of identified failure modes common to both teams^a^

Causes of failure modes identified by both teams	Relevant failure mode/s number/s (failure mode numbers are according to Table 2.)
Overcrowded medication counters	1, 2, 3, 4, 9, 12, 13, 14, 15, 16, 17, 18, 19, 20, 21, 22, 23, 24, 25, 26, 27, 28, 29, 31, 32, 33, 34, 35, 41
Pharmacists working long hours without a break due to inadequate staff	5, 6, 7, 8, 12, 13, 14
Unclear prescriptions	5, 6, 7, 8, 35
Improper arrangement of dispensing tables	10, 11
Not rechecking the dispensed medications	12, 13, 14, 28, 29, 31, 32
Negligence/poor attention by pharmacist	10, 12, 13, 14, 16, 17, 18
Environmental distractions and interruptions	22, 23, 31, 33, 42
Improper/ unclear labels attached to the pre-packed medication packs	10
Poor communication with patients	9, 19, 20, 35, 40
**Effects of failure modes identified by both teams**	
Patient receiving wrong medication	
Patient receiving wrong dose of medication	
Patient receiving wrong quantity of medication	
Patient taking medications incorrectly due to unclear instructions (verbal and/or written)	
Patient does not achieve the intended therapeutic outcome which will lead to loss of medication adherence	
Patient does not receive all required medications	
Patient receives unnecessary medications (e.g. omitted medications/ medications prescribed in a previous visit)	
Another healthcare professional will not able to identify the medications taken by the patient if allergy develops or treat other health condition when medication name is not indicated on the label	
Patient medication histories and hospital copy of the patient’s prescription are lost/misplaced if medications were dispensed from the wrong pharmacy counter	

^a^FMEA spread sheets are available as supplementary material for further details

Having overcrowded medication counters was stated as a cause for 57 failure modes by both teams. In addition to causes commonly identified by both teams, Team B identified, pharmacists not adhering to a uniform method of medication labeling as a cause for unclear instructions; Poor communication among pharmacists as a reason to miss notifying about out of stock medications to patients; Inadequate supervision of the medication repacking process carried out by support staff (non-pharmacists) leading to medication errors.

### Step 4 – giving a numerical value (scoring) for severity, frequency and detectability of each failure mode and calculating the risk priority number (RPN)

The highest RPN given by Team A was 40 which was for the failure mode, “Counting the wrong amount of medication” when filling medications in the dispensing process. The lowest RPN value by Team A was two which was for “Use of unclear printed information material (patient information leaflets)” when dispensing medications with verbal instructions and “Failing to document dispensing accountable medications”.

The highest RPN given by Team B was 24 which was scored for three failure modes; 1) Mixing up two prescriptions given by one person when checking the prescription (If one person comes to collect their own medications and of another), 2) Failing to identify overdoses and interactions when checking the prescription, and 3) Incomplete labeling when labeling and assembling medications. The lowest RPN value for Team B was also two. The two failure modes with RPN of two were for 1) Unavailability of printed information material (patient information leaflets) when dispensing medications with verbal instructions and 2) Adding the hospital copy of the prescriptions into the wrong storage box after documentation. RPN values assigned by both teams for each failure mode are shown in Table 2.

### Step 5 – suggesting corrective actions for prioritised failure modes

Team A prioritised 36 failure modes and Team B prioritised 30 failure modes to be discussed for corrective action. Some of the major suggestions were applicable for more than one failure mode. Team A suggested that failure modes such as misidentification of clinics and unclear prescriptions could be resolved with the introduction of a computerised prescribing system, and bar code identification of patients. Other suggestions by Team A for 22 failure modes were to redesign the dispensing area with patient waiting facilities, and to limit one patient per one dispensing counter at a time.

The major suggestion by Team B for seven failure modes was to reorganise the dispensing process where dispensed medications could be rechecked by at least two pharmacists (having more than one pharmacist involved in dispensing to one patient). Other solutions suggested by them were to increase communication with patients, establish a separate patient counseling unit with a pharmacist, display maximum doses and serious interactions of commonly used medications to be easily viewed by the dispensing pharmacists, display the list of out of stock medications and regularly updating the list, hang an alert label on containers with short expiry medications three months prior to the expiry date, and to redesign the medication repacking process to be carried out under the supervision of a pharmacist.

Commonly suggested corrective measures were increasing the awareness of pharmacists on patient safety and responsibility of pharmacists through continuous education, redesigning the labels of pre-packed medication packs with a colour code for identification, rearranging all dispensing shelves in a uniform manner and separating look-alike containers. Increasing the number of pharmacists was suggested as a corrective action by Team A for 28 of 48 failure modes, and by Team B for four failure modes.

Feedback results reported a positive feedback from almost all the team members from both groups. Feedback results are shown in Table 5.

**Table 5:** Feedback of team members

Feed back	Total number of participants (***N*** = 13)	
	Agreed frequency (N)	Agreed percentage (%)
I feel that this method (FMEA group discussions) is an effective method to analyse the dispensing process.	13	100
The discussions were interesting to me.	13	100
The discussions made me to think more deeply on my day today practice and patient safety.	12	92.3
The discussions allowed us to share the experiences and ideas of other colleagues.	12	92.3
I feel that this method is a time-wasting procedure.	1	7.7
I feel that the scoring method and failure mode identification depends on experience of individuals.	12	92.3
I think that identified failure modes and solutions made for our setting can directly apply to any other setting (another hospital), without any change.	5	38.5
I recommend that this method can be applied to analyse other areas of hospital pharmacy such as indoor dispensing, stores management.	10	76.9

## Discussion

This study aimed at using FMEA to prospectively identify failure modes, possible causes, and related corrective action, to improve the safety of the dispensing process at a selected tertiary care hospital in Sri Lanka. A total of 90 failure modes were identified by the two FMEA teams. They identified overcrowded medication counters, long working hours, unclear prescriptions, distracted working environment, not rechecking the dispensed medications, negligence of the pharmacists, communication issues, improper dispensing tables, and improper labeling as common causes for failures which could result in patients receiving wrong medications and/or medication doses, and in turn lead to poor medication adherence. Teams proposed the need for redesigning dispensing counters, dispensing shelves and medication labels to improve medication safety in the dispensing process, while supervision of the medication repacking process by a pharmacist, including two or more pharmacists in the medication dispensing process, and establishing a separate patient counseling unit with a dedicated pharmacist were prioritised as process improvements.

FMEA was conducted in two teams to minimise the disruption to routine dispensing services at the study hospital and was successfully completed by both teams. Most FMEA studies found in the literature proceeded with one team [2, 12, 19–21, 24, 27, 31, 34, 36]. Shebl et al., [15] reported a FMEA study conducted using two teams in two settings. Some other researchers also used more than one group for scoring of failure modes [16] and to represent multiple units of a single setting [38].

Both teams mapped the dispensing process in a similar manner except when Team A identified two pathways of medication assembling while Team B identified this division at the sub process level. However, the dispensing process map identified by our teams is similar to those mapped by others [12, 27] except where the step on rechecking medications before dispensing is missing in ours. Nevertheless, pharmacists in both teams identified this missing step as a cause of error, and Team B even suggested redesigning the dispensing process to include a rechecking step by a second pharmacist.

Among all 90 failure modes, 69 failure-modes were independently identified by both teams indicating the suitability and reliability of the FMEA process in diagnosing critical issues in a system. Failure modes identified by both teams such as, failure to identify prescription errors, incomplete and/or incorrect medication labeling, and insufficient verbal information given to patients were also identified by other researchers [12, 27]. Similar to our findings, a study conducted in a community pharmacy in Serbia [12] stated that dispensing wrong medication/dose/quantity are also possible failure modes.

Causes of failure modes documented in this study were also consistent with studies done worldwide. FMEA studies conducted on areas such as chemotherapy [18, 43], medication prescribing, prescription validation and dispensing for in-patients [36] and on medication administration [15] in countries such as the Netherlands, China, Spain and the United Kingdom reported work overload due to inadequate staff as a major cause of medication errors which is similar to our findings. Like in this study, communication issues between healthcare professionals and patient was also reported as a cause of error by many others [2, 12, 20, 22, 27, 34]. Environmental distractions, illegible handwriting of prescriptions and/or labels, knowledge deficit of healthcare professionals, and lack of awareness of healthcare professionals acknowledged as causes in this study were also shared by many other researchers [12, 15, 17, 20, 22, 27, 33, 34].

Interestingly, corrective action suggested by team members in this study were also similar to those reported by other FMEA studies. Incorporating modern technology such as computerised prescription order systems, and bar code identification of patients [12, 15, 17, 24, 27, 33, 38], improving communication strategies, and double checking of any healthcare process [12, 15, 18, 20, 21, 24, 27, 33, 38, 40, 43, 44] were the most commonly highlighted solutions by many. A systematic review [45] conducted to assess the effect of new technologies such as barcode identification, computerised prescriptions and automated dispensing devices reported a reduction in medication errors after implementing new technology although they recommend to conduct a cost-benefit analysis on using new technology. Another study has shown that barcode assisted medication administration has a significant impact on reducing medication errors than manual double check [46]. Participants from Team B of the present study also suggested double checking dispensed medications, which should be re-considered due to the practical issues that may arise from the need of more human resources and some prevailing uncertain discussions among the scientific community on the double checking procedure [47, 48].

Although the inability to generalise results is an inherent limitation of FMEA, there were marked similarities of failure modes, causes, and solutions, of medication errors identified among different studies using this proactive tool across a variety of healthcare settings. Thus, we believe that the findings of this study too will be applicable to similar healthcare settings. However, we must acknowledge the subjective nature of FMEA studies which was apparent when assigning RPN values to failure modes by Teams, A and B. The mathematical accuracy of calculating RPN values has been a concern for other researchers as well [49] and is a known limitation of FMEA.

Failing to conduct a second FMEA after implementing corrective action is a limitation of this study. Subjectivity and inability to generalise the results are other limitations in this study as also reported by past studies [15, 49]. Furthermore, there was some concern regarding the reliability of prioritising failure modes, as the two groups assigned different RPN values for the same failure mode in some instances. However, this model provides evidence that FMEA can be successfully used to identify possible failure modes of the dispensing process in out-patient care of hospitals. Conducting an FMEA makes pharmacists more aware of possible failure modes as they are personally involved in this activity. Feedback obtained from team members also revealed that this process helped them to think seriously on possible failure modes possible in day today practice and provided a good platform to share experiences among fellow colleagues.

## Conclusions and practical implications

This study depicts a model of successfully using FMEA to identify and prioritise possible failure modes, causes and possible corrective actions, of the dispensing process through active involvement of pharmacists. Two FMEA teams identified 90 possible failure modes in the dispensing process, their causes and effects.

Conducting a proactive assessment such as FMEA helps pharmacists to be more vigilant and be actively involved in minimising medication errors. As a result of this FMEA study, corrective action which could be implemented easily such as improving dispensing labels with colour codes, incorporating the quantity of medications on the dispensing label, and re-organising of all dispensing tables were initiated immediately. General suggestions to improve medication safety of the dispensing process (highlighted during the study) were brought to a discussion table with the management of the Department of Pharmacy including the establishment of a separate medication re-packing unit and redesigning of dispensing counters serving one patient at a time. Further, a study was initiated to assess the medication safety of the medication re-packing process.

Finally, we suggest that this effort could be used as a guide by other similar institutes in order to achieve a safer medication dispensing system and to offer better pharmaceutical care with minimum hazards.

## Data Availability

All data generated or analysed during this study are included in this published article.
